# Teaching case 2-2019: Macrophagic scavenging of Aβ 

**DOI:** 10.5414/NP301175

**Published:** 2019-02-01

**Authors:** Ellen Gelpi, Sigrid Klotz, Alexandra Lang, Elisabeth Stögmann, Gabor G. Kovacs

**Affiliations:** 1 Institute of Neurology, Medical University of Vienna, Vienna, Austria,; 2 Neurological Tissue Bank of the Biobanc-Hospital Clinic-IDIBAPS, Barcelona, Spain, and; 3 Department of Neurology, Medical University of Vienna, Vienna, Austria

**Keywords:** Aβ peptides, intracellular, scavenging, macrophages, microglia, astrocytes

## Abstract

No abstract available.

The extracellular accumulation of Aβ peptides (generated by enzymatic cleavage of the amyloid precursor protein by β- and γ-secretases) in form of dense amyloid plaques in the brain is considered one of the histological hallmarks of sporadic and genetic Alzheimer’s disease (AD) as well as of Down syndrome. These deposits disrupt the surrounding neuronal processes in the grey matter neuropil and generate dystrophic neurites (= neuritic plaques) and neuronal dysfunction. It is known that Aβ may have different assembly states: monomers, dimers, trimers that arrange in protofibrils and fibrils, or globulomeres and unstructured oligomers into fibrils, with different physiopathological consequences, oligomers most likely with being the most toxic species [1]. Diffuse Aβ deposits are observed in the aging brain throughout the cortical and subcortical grey matter and in subpial regions, especially in areas affected by cerebral amyloid angiopathy. Whether diffuse deposits progressively condensate and eventually evolve into cored or dense plaques is a matter of debate [2]. Moreover, the presence of intracellular Aβ peptides has been widely discussed, especially whether its detection depends on technical issues (e.g., tissue pretreatment and fixation strategies) [3, 4] and whether it represents a physiological or pathological state. But it is increasingly suggested, especially from animal models, that intracellular Aβ accumulation may represent an early phenomenon in the pathogenic cascade of AD [5, 6, 7, 8, 9], that would lead to early neuronal and synaptic dysfunction [10]. 

Microglia, as part of the innate immune system of the CNS, could be considered to be one of the cellular elements responsible for environmental supervision, and as such, capable of scavenging abnormal protein aggregates including Aβ in its activated, phagocytic, and proinflammatory state. At the same time, the release of cytokines and other inflammatory mediators by microglia contributes to Aβ oligomerisation, cross-seeding and aggregation [11], and to synaptic damage. Concurrently, Aβ peptides are able to activate microglia, which in turn generates a vicious cycle between microglia and Aβ [12]. 

Here we show that macrophages, coming from peripheral blood, are also capable to phagocytose extracellular Aβ peptides, either diffuse, primitives, or cored plaques [13]. This can be well identified in areas affected by an ischemic infarction, as shown in Figure 1A1, A2, and A3, where macrophages are filled with Aβ peptides (Figure 1A3), suggesting their digestion and degradation. 

As already described before [15] we show in the lower figure panel (Figure 1B1, B2, B3) early diffuse cloudy extracellular deposits of Aβ peptides in cerebral cortex surrounding neurons and astroglial cells that accumulate Aβ within their cytoplasm (Figure 1A1) [15]. This is frequently observed in postmortem brains of elderly subjects. In contrast, while extracellular plaques condensate (Figure 1A2, A3), the intracellular Aβ component progressively disappears or gets – at least – less evident. 

Wisniewski et al. [16] already observed in ultrastructural studies that Aβ peptides in macrophages are located in lysosomes, suggesting phagocytosis, while in microglia they were observed in the reticulum, suggesting production. Some studies have suggested that microglia scavenging of Aβ peptide is less efficient than that by macrophages [13]. However, it is unclear whether peripheral macrophages can easily infiltrate the brain in AD patients. Efficiency of microglia clearing has been experimentally increased in a proinflammatory state. Therefore, disrupting the vicious cycle between Aβ and microglia and enhancing immunocompetency in the CNS regulating the imbalance between protection and toxicity, might be an important therapeutic approach in AD and other Aβ-related conditions. 

## Funding 

The authors declare no financial disclosures. 

## Conflict of interest 

The authors declare no competing interests. 

**Figure 1.**
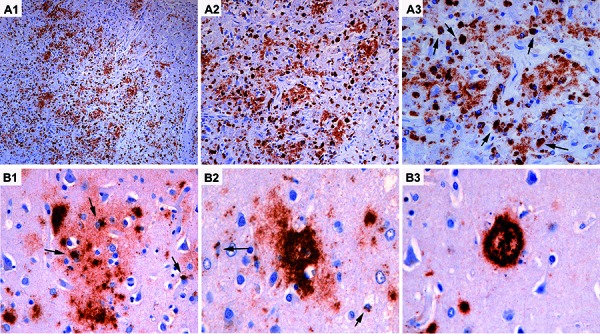
Scavening and states of Aβ deposits. Aβ scavening: A1 – A3: Abundant macrophagic activity in an area of ischemic brain infarction. Macrophages show abundant Aβ-positive material in their cytoplasm (A2, A3), representing internalization and degradation of Aβ peptides (arrows in A3). Different states of Aβ deposits: B1: Diffuse deposits with centrally located neurons and especially (astro)glial cells accumulating Aβ peptide in the cytoplasm; B2: Aβ deposits in form of primitive plaques and B3: in cored plaques are less associated with intracellular Aβ (arrow in B2).
